# Treatment of open tibial diaphyseal fractures by external fixation combined with limited internal fixation versus simple external fixation: a retrospective cohort study

**DOI:** 10.1186/s12891-019-2679-9

**Published:** 2019-07-03

**Authors:** Zi-Chen Hao, Yan Xia, De-Meng Xia, Yun-Tong Zhang, Shuo-Gui Xu

**Affiliations:** Department of Emergency, Trauma Center, Changhai Hospital, The Second Military Medical University, No 168, Changhai Road, Yangpu District, Shanghai, 200433 People’s Republic of China

**Keywords:** Open tibial diaphyseal fractures, External fixation, Limited internal fixation, Combined fixation

## Abstract

**Background:**

The treatment of open tibial shaft fractures is challenging. External fixation (EF) is comparatively safe in treating these open injuries, meanwhile it has the advantages of easy application, minimal additional disruption, and convenient subsequent soft tissue repair. Nevertheless, its application is accompanied by a series of problems in alignment and bone healing. Therefore, limited internal fixation (LIF), such as cortical screws, has been used based on the external fixator for better therapeutic effect. The aim of this study is to compare the outcomes of EF combined with LIF and simple EF in the management of open tibial shaft fractures, evaluating the efficacy and safety of using the combined technique in treating such fractures.

**Methods:**

From January 2012 to December 2016, patients with open tibial shaft fractures treated with EF with or without LIF augmentation were identified. A total of 152 patients were included in the analysis, and there were 85 patients in the simple external fixation group and 67 patients in the EF-LIF group. General assessment indicators included the direct cost of hospitalization and the times of first surgery, full weight bearing, and complete union. Infections and complications in union or limb alignment were compared as primary outcomes. Additionally, the number of patients who changed the fixation system for various reasons were analysed.

**Results:**

Effective follow-up of all participants for statistical analysis was obtained. The follow-up time averaged 17.15 months (range: 12.00 to 24.00 months) in the EF group and 16.20 months (range: 12.00 to 19.00 months) in the EF-LIF group. Combined fixation provided shortened time to bear full weight and achieve complete bone union, while requiring additional first surgery time. No significant difference was found in infection rates or direct cost of hospitalization. Delayed union and non-union in the EF-LIF group were significantly decreased (20.9% versus 40.0, 1.5% versus 14.1%, *p* < 0.05). In limb alignment, patients with combined fixation exhibited reduced malreduction, loss of reduction, and malunion. In terms of secondary fixation, the EF-LIF group showed a markedly lower incidence (5.8% versus 34.1%, *p* < 0.001).

**Conclusion:**

Compared with simple EF, combined fixation is an effective and safe alternative for management of open tibial diaphyseal fractures. It provides superior initial reduction, better stability and decreases the risk of inferior alignment and delayed union without increasing the risk of infection.

## Background

Open tibial fractures account for over 40% of all open fractures and are frequently accompanied by significant damage to soft tissues, including skin, muscle, and neurovascular structures [1]. These fractures are especially prone to serious complications, such as infection, malunion, and non-union, adding to the incidence of readmission and reoperation [2, 3]. Thus, a timely and appropriate treatment protocol that involves thorough debridement, accurate reduction, early and stable fixation, repair of soft tissues, and administration of antibiotics has been widely accepted for reducing complications and increasing the chances of bony union [2, 4].

The fixation methods for open tibial diaphyseal fractures have evolved over the years but remain controversial [4–6]. Damage control orthopaedics (DCO) with external fixation followed by definitive internal fixation with nailing or plating is a popular strategy for significantly decreased complications [7]. However, the secondary fixation procedure causes economic, physical, and psychological burdens, making the strategy less than ideal [7–9]. Thus, external fixation is an alternative as the definitive fixation in some cases such as improper conditions of soft tissues or patients’ non-compliance for the staged surgeries. Nevertheless, based on relevant studies, external fixation as a definitive treatment should warrant more attention for possible issues related to pin-track infection, unsatisfactory alignment, and poor union, leading to unplanned secondary fixation procedures and consequent additional burdens to patients in physiology and economy [5, 10–14].

To improve the performance of external fixation for treating such fractures, additional minimal internal implants, such as cortical screws and Kirschner wires, have been recommended [14–18]. Nevertheless, the combined technique brings the concern for increased infection risk due to metal implants, especially in these open injuries. Relevant studies have been rarely reported, and there is a lack of reliable data regarding treatment outcomes. Therefore, the present study retrospectively compared the outcomes of the combined technique with simple external fixation to evaluate the effectiveness and safety of combined fixation in treating open tibial diaphyseal fractures.

## Methods

### Study design

All open tibial fractures treated with simple EF (Hoffmann II External Fixation System, Stryker Corporation, Kalamazoo, Michigan, USA) or EF augmented by LIF, including cortical screws and the Ni-Ti arched shape-memory connector [19–22] (ASC, Swan Biological Memory Medical Devices Co., Ltd., Huzhou, Zhejiang Province, China), at our trauma centre were identified through the hospital’s electronic patient files system in the period between January 2012 and December 2016. This study was approved by the institutional review board of our hospital. Medical records and images were retrieved to exclude patients who met the following criteria: [1] aged younger than 18 years at the time of injury, [2] fractures located within 5 cm of the proximal or distal articular surface, [3] did not undergo a timely operation within 8 h after trauma, or [4] injuries of Gustilo-Anderson grading IIIB or IIIC. Ultimately, 85 and 67 patients were included in the EF group and the EF-LIF group, respectively, for this study. The demographic characteristics of patients in each group are listed in Table 1. Both groups were comparable in terms of every listed general characteristic.

**Table 1:** Patient demographic

	EF (*n* = 85)	EF-LIF (*n* = 67)
Age (years)		
Mean (range)	45.31 (24 to 63)	42.17 (26 to 65)
Gender		
Male, n (%)	55 (64.7)	41 (61.2)
Female, n (%)	30 (35.3)	26 (38.8)
Fracture type (AO system)		
A, n (%)	34 (40.0)	28 (41.8)
B, n (%)	32 (37.6)	26 (38.8)
C, n (%)	19 (22.4)	13 (19.4)
Gustilo-Anderson grading		
I, n (%)	24 (28.2)	21 (31.3)
II, n (%)	45 (53.0)	31 (46.3)
IIIA, n (%)	16 (18.8)	15 (22.4)

### Surgical technique

**Fig. 1:**
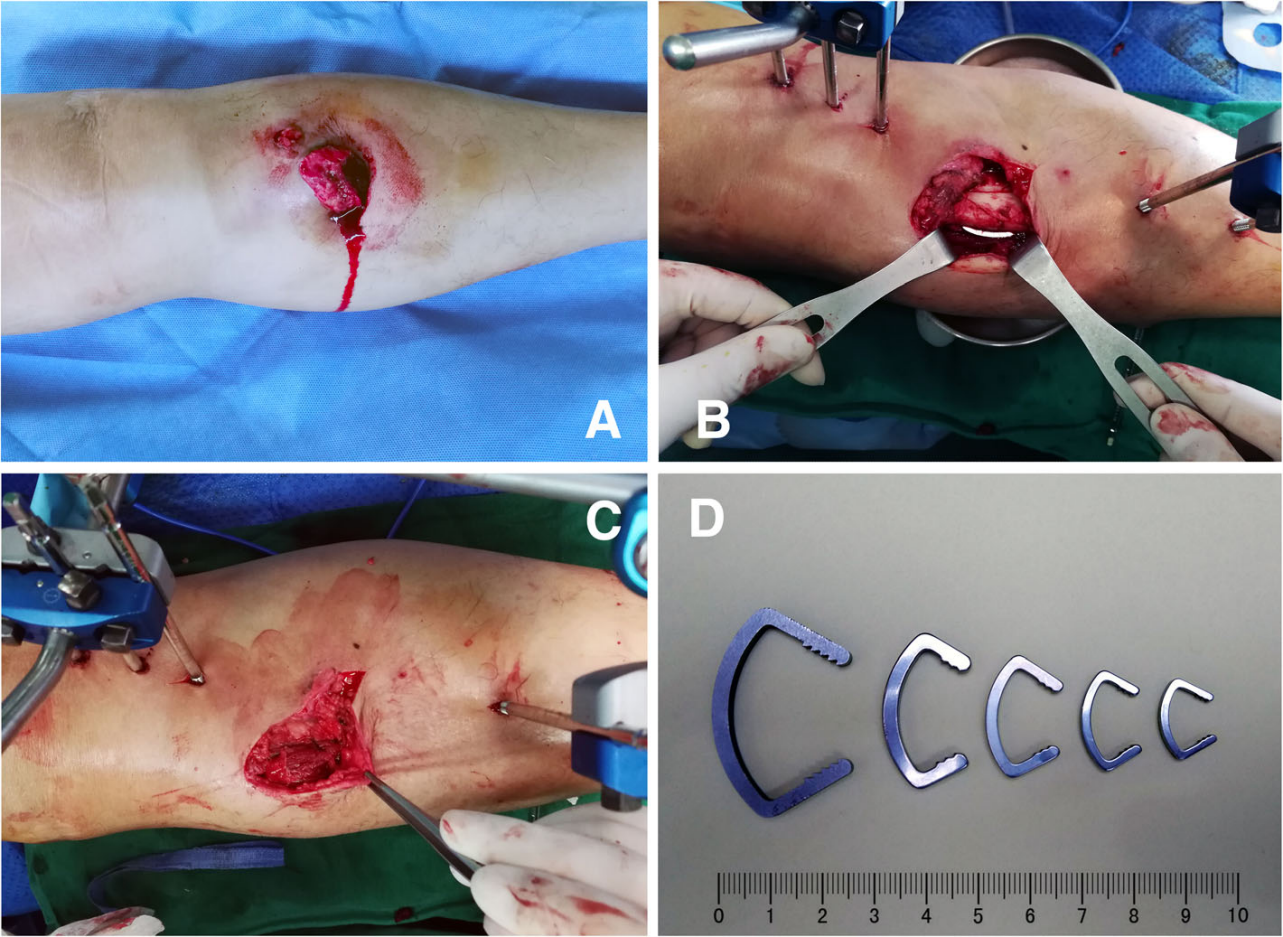
Brief intraoperative procedures for a 44-year-old male with grade II open tibial shaft fracture who accepted external fixation combined with limited internal fixation. **a** The initial injured limb. **b** Surgical fixation with an external fixator and limited internal apparatus of a Ni-Ti arched shape-memory connector (ASC). **c** Sufficient soft tissue coverage after placing the internal implants. **d** Different models of the ASCs

**Fig. 2:**
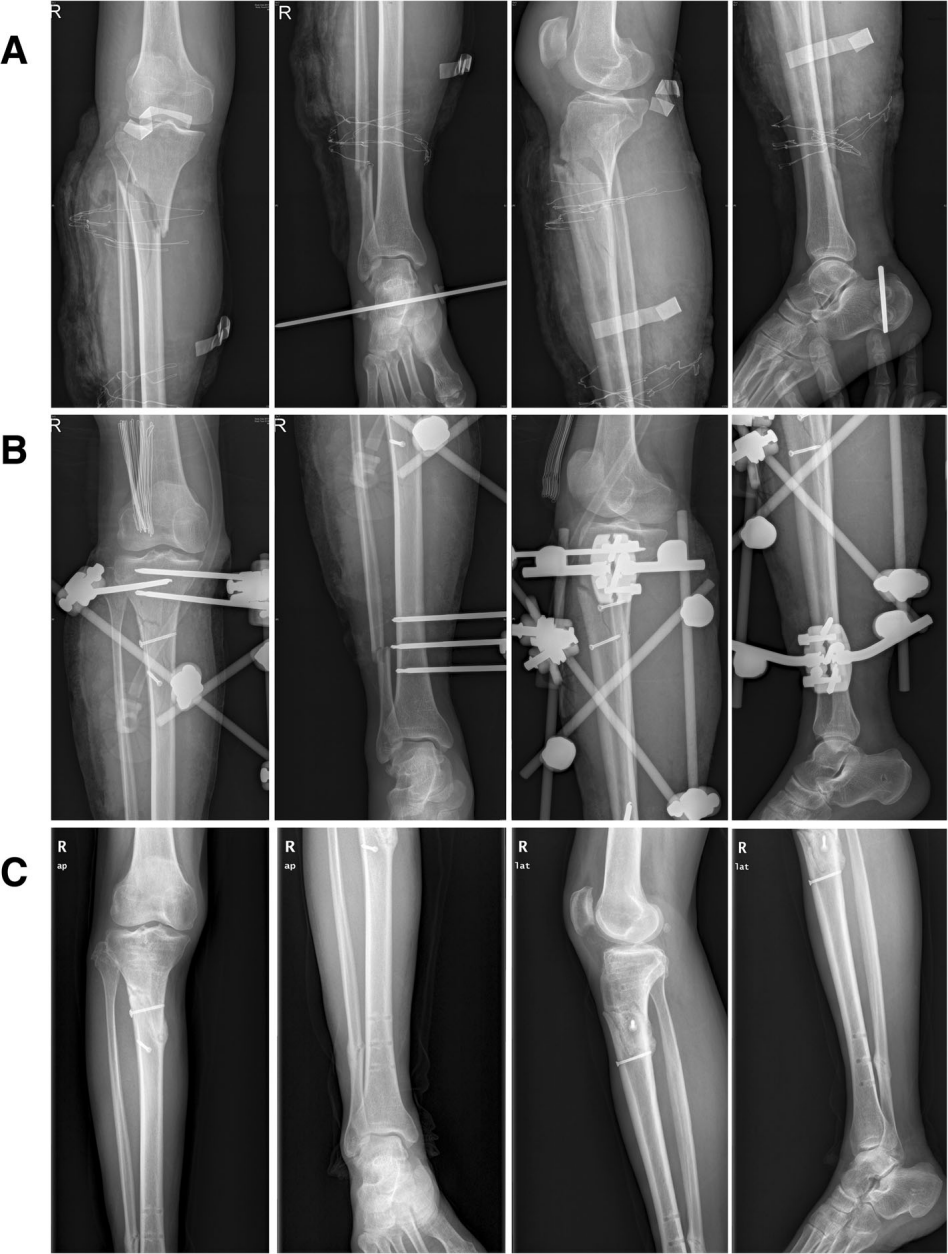
An illustrative case treated with external fixation combined with cortical screws. **a** Preoperative radiographs of the injured limb. **b** The initial postoperative radiographs following application of an external fixator and cortical screws. **c** Radiographs at final follow-up (12 months after the initial operation)

**Fig. 3:**
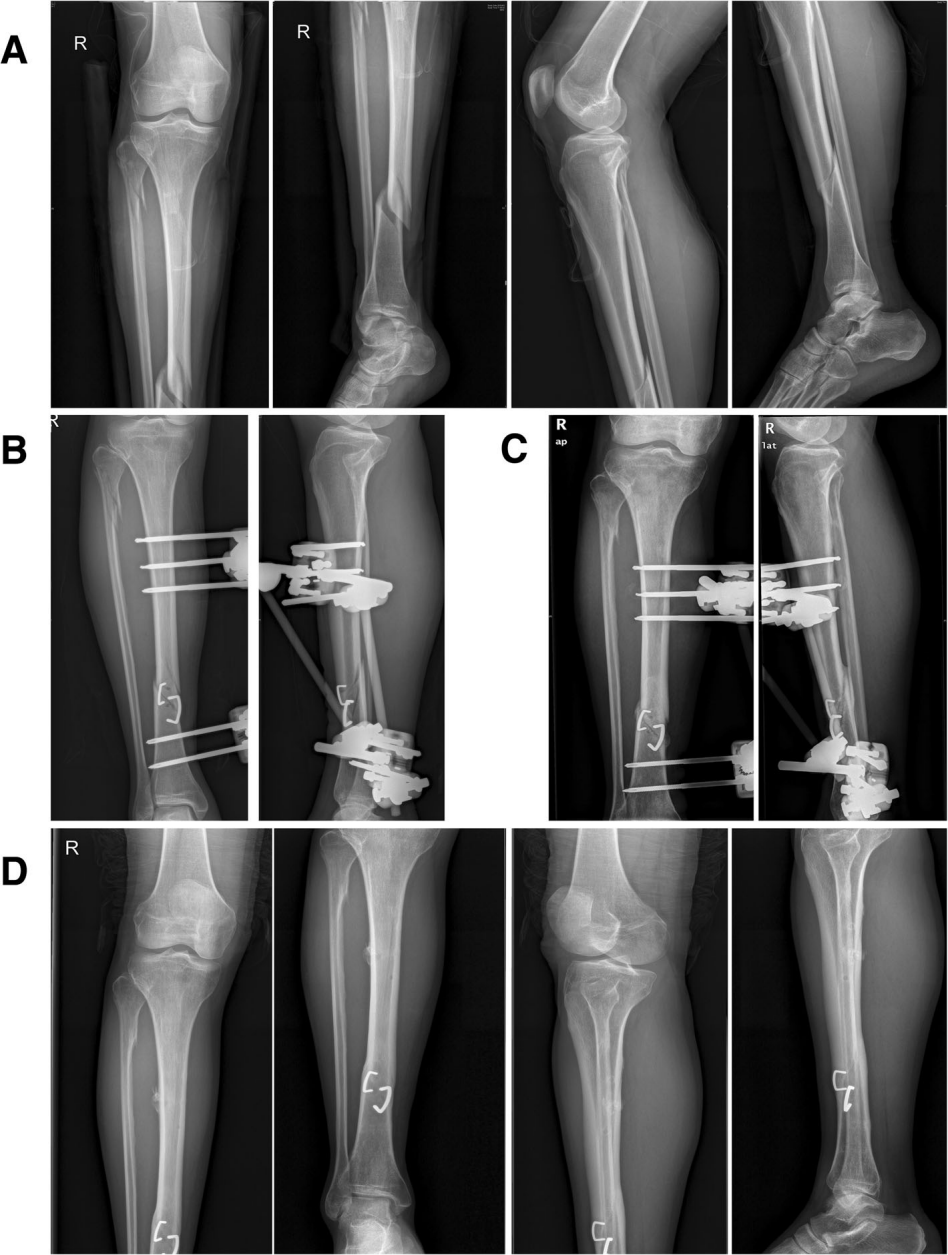
An illustrative case treated with external fixation combined with ASCs. **a** Preoperative radiographs of the injured limb. **b** The initial postoperative radiographs following application of an external fixator and ASCs. **c** Radiographs 6 months after from the initial operation. **d** Radiographs at final follow-up (17 months after the initial operation)

All surgeries were performed by two senior orthopaedic surgeons on the same surgical team. Patients were operated on within 8 h after trauma under coverage of broad-spectrum antibiotics. Thorough irrigation and debridement were performed to eliminate all contaminants, as well as highly contaminated or necrotic soft tissue. After temporary reduction with two or more Kirschner wires, one to two cortical screws or ASCs were placed in a site with sufficient coverage of soft tissues in the combined fixation group. In general, ASCs were used for transverse or short oblique fracture patterns, and cortical screws were used for long oblique or spiral fracture patterns. A more detailed instruction of the structure and working principles for the ASC can be reviewed in previous reports [20, 22]. Subsequently, three 5.0-mm Schanz screws were inserted into the distal and proximal bone segments. Frames of the Hoffmann II External Fixation System were assembled in the shape of a triangle with proper tightening. Afterwards, intraoperative radiographs were obtained to evaluate the final reduction. Next, the wound was sutured primarily or secondarily based on specific circumstances. Finally, involvement of a plastic and reconstructive surgeon with split-thickness skin graft or a flap was performed as early as possible in the subsequent treatment process. Intraoperative procedures are shown in Fig. 1, and related radiographs of two acute fracture cases treated with the combined technique are shown in Fig. 2 and Fig. 3.

### Postoperative management

Commonly, one month after the initial operation, follow-up radiographs were obtained to evaluate the progress of the fracture union. Subsequently, gradual weight-bearing was permitted. Subsequently, patients returned for both clinical and radiological assessment every 2 months until the fracture united. During this period, full weight bearing was allowed when an adequate bridging callus was visible on radiographs. The union of the fracture was defined as the presence of a bridging callus in at least three cortices based on the radiograph, while meeting the requirement of no pain or tenderness over the fracture zone [23]. The external fixation system was removed, as an outpatient procedure, after complete union of the fracture. Related complications and significant data, such as time to full weight bearing and time to complete bony union during follow-up, were recorded. An effective follow-up of all participants for statistical analysis was obtained. The follow-up time averaged 17.15 months (range, 12.00 to 24.00 months) in the EF group and 16.20 months (range, 12.00 to 19.00 months) in the EF-LIF group.

### Outcomes

Four general indicators, including direct cost of hospitalization, first surgery time, time to full weight bearing, and time to complete union were compared between the groups. In addition, the incidences of three major complications were also compared.

1. Presence of infection—Infection was subdivided into pin-track infection, superficial wound infection, and deep tissue infection. A pin-track infection was inflammation around the pin-track. A superficial wound infection was one located in the initial or operative wound that was above the fascia, with erythema and tenderness. A deep infection was defined as infection involving deeper tissues, such as muscular fascia and bone.

2. Time to union—Normal healing was defined as healing within 6 months, and delayed union was regarded as healing after 6 months. A fractured bone that did not completely heal within 9 months of injury, as well as showing inapparent progression towards healing over three consecutive months on serial radiographs was characterized as non-union [12, 13, 24].

3. Limb alignment—Limb alignment was determined by angulation, shortening, and rotation. Angulation was measured in the coronal or sagittal plane. Shortening was determined by clinical comparison with the contralateral leg. Rotational malalignment was measured in both lower extremities clinically as the thigh-foot angle (TFA) and when suspicions remained, a determination was made by CT. Unsatisfactory alignment was defined when one of the following criteria was met: [1] shortening of 1 cm or more; [2] varus or valgus angulation of 5 degrees or more; [3] anterior or posterior angulation of 10 degrees or more; or [4] rotational malalignment of 10 degrees or more compared with the contralateral leg [13]. All unsatisfactory cases were recorded as malreduction, loss of reduction, or malunion. Of these, malreduction was defined as unsatisfactory reduction in the first operation. Loss of reduction occurred during the follow-up period in patients with a successful surgical reduction. Malunion was regarded as the final deficient alignment of the fracture.

All statistical analyses between groups were performed using the x^2^ test for categorical and ordinal variables and Student’s t-test for continuous variables (SPSS 19.0 statistical software). Significance tests were two-sided at the 5% significance level.

## Results

### Utilization of the limited internal fixation

For the limited internal implants, ASCs were applied in 30 patients, cortical screws were used in 27 patients, and a combination of ASCs and cortical screws were used in 10 patients.

### General indicators

The results of general assessment indicators are listed in Table 2. In terms of direct cost of hospitalization, there was no significant difference between patients treated with the two different protocols. However, after excluding the patients who underwent a secondary operation for changing fixation, the average direct cost of hospitalization was somewhat lower in the EF group. Approximately half an hour was saved for the first surgery with simple EF compared with combined fixation. Nevertheless, time to full weight bearing and complete bony union were both longer in the EF group.

**Table 2:** Comparison of general assessment indicators between two groups

General assessment indicators (units)	EF (n = 85) (range)	EF-LIF (n = 67)	*p* value
Total cost (USD)	9188.53 (2303.38 to 19,036.03)	8193.68 (4068.82 to 15,442.94)	0.062
Patients without changing fixation	7164.26 (*n* = 56) (2303.38 to 9877.50)	8096.91 (*n* = 63) (4068.82 to 15,442.94)	0.041
First surgery time (hours)	1.20 (0.43 to 2.12)	1.45 (0.68 to 2.17)	0.002
Time to full weight bearing (months)	4.94 (3.10 to 6.83)	3.41 (2.73 to 4.27)	< 0.001
Time to complete union (months)	6.91 (4.60 to 14.13)	5.84 (4.22 to 10.25)	< 0.001

### Primary complications

Table 3 shows the comparison of major complications for the two strategies. No significant difference was observed in terms of pin-track, superficial wound, or deep tissue infections. All patients with these infections recovered after appropriate treatment, including removal of internal hardware, repeated debridement, or administration of antibiotics. In terms of bone union, the incidence of delayed union and non-union were significantly higher in the EF group. Patients diagnosed with non-union obtained recovery after bone grafting and reamed tibial nail fixation. In terms of limb alignment, patients treated with simple EF presented significantly higher rates of malreduction, reduction loss, and malunion.

**Table 3:** Comparison of major related complications between two groups

Complications	EF (n = 85)	EF-LIF (n = 67)	*p* value
Infection			
Pin-track infection, n (%)	36 (42.4)	30 (44.8)	0.765
Superficial wound infection, n (%)	12 (14.1)	10 (14.9)	0.888
Deep tissue infection, n (%)	5 (5.9)	4 (6.0)	1.000
Delayed union, n (%)	34 (40.0)	14 (20.9)	0.012
Non-union, n (%)	12 (14.1)	1 (1.5)	0.013
Malreduction, n (%)	14 (16.5)	3 (4.5)	0.038
Loss of reduction, n (%)	25 (29.4)	4 (6.0)	0.001
Malunion, n (%)	15 (17.6)	2 (3.0)	0.010
Secondary operation of changing fixation system, n (%)	29 (34.1)	4 (6.0)	< 0.001

In addition, patients undergoing additional surgery to change the fixation system for diverse reasons were recorded. The findings are also listed in Table 3. Overall, the EF group had a far higher incidence of changing fixation than did the EF-LIF group (34.1% versus 6.0%, *p* < 0.001). Of the total 29 patients in the EF group, 13 patients underwent an operation to improve alignment as a result of malreduction or reduction loss, 12 patients underwent an operation because of non-union, and 4 patients underwent an operation for reaching better union due to slow healing. In contrast, there were only 4 cases in the EF-LIF group, of which two were ascribed to malreduction, one was due to loss of reduction, and another was for non-union.

## Discussion

Simple external fixation used to treat open tibial shaft fractures is accompanied by a high incidence of complications in terms of reduction, alignment, and bone healing [5, 10–14]. In a randomized and prospective study from Holbrook et al. [13], 28 open tibial shaft fractures treated with simple external fixation had a fairly high rate of pin-track infection (21%), reduction loss (11%), malunion (36%), delayed union (21%), and non-union (11%). Henley et al. [12], in a report on 70 patients with type II, IIIA, and IIIB open fractures of the tibial shaft, noted 17.1% delayed union, 4.3% non-union, 50.0% pin-track infection, and 31.0% malunion after external fixation. Moreover, 17.0% fractures underwent a change in fixation due to delay in healing or loss of reduction [12]. Patients with simple external fixation also exhibited similarly high complication rates in our study. The incidence of changing the fixation system was as high as 34.1%, indicating the potential problems of simple external fixation in treating open tibial shaft fractures [5, 10–14]. The biomechanical limitations of external fixation for adequate reduction and stability and premature removal of the fixator in the healing process were believed to be the leading causes [5, 10–14].

Thus, in order to improve the biomechanical stability of the external fixation system, additional minimal internal apparatus, such as cortical screws, have been recommended for the management of open tibial diaphyseal fractures [14–18]. As early as 1983, Spiegel et al. [17] adopted the combined technique in several patients and believed that it could decrease pin-track infection, accelerate early weight-bearing, and enhance healing. However, detailed statistical data were not given in their report. Subsequently, Bach et al. [15] conducted a prospective study of 59 patients with grade II or III open tibial shaft fractures to compare internal and external fixation. In the EF group, minimal internal fixation was also used to obtain and maintain anatomic reduction in 12 patients. Although they did not analyse the patients treated with combined fixation independently, the EF group showed decreased malunion (10%), changing fixation (7%), and non-union (none) [15]. Despite some investigators advocating the combined technique in their reports [14–18], no comparatively large series study exclusively aimed at open tibial shaft fractures has been reported. In our study, 67 patients with the combined technique were retrospectively analysed, providing relatively new and valuable data in this field.

First, the two therapies presented similar infection rates, indicating that the additional LIF did not lead to increased infection risks, even in Gustilo-Anderson grade II or IIIA fractures, as long as thorough debridement and adequate soft tissue coverage were performed. A similar conclusion can be obtained from the experience of treating other open injuries, especially intra-articular tibial fractures [25–27]. Along with external fixation, limited internal fixation can offer sufficient stability and may decrease the incidence of subsequent reduction loss. The maintenance of the favourable reduction that is conducive to fracture healing can be inferred from the decreased number of union complications and the shorter time to full weight bearing and complete union. As a result, accelerated return to functional exercise and prompt return to normal work and life can be obtained.

However, some concerns are also proposed with this combined technique. First, without taking changing fixation into consideration, patients treated with the combined technique required additional surgical time and expenditure on average; this should be noticed when the patients are in poor health or poor economic condition. Moreover, although the internal implants were placed through the intrinsic wound or a necessary minor incision (rarely causing further disruption of the periosteum or compromise of the blood supply), additional disruption to surrounding soft tissues may occur.

In addition, there are some inherent limitations of external fixation. A high pin-track infection rate is the most common problem. The pin-site infection rate was as high as 44.8% in our study, but this did not result in an increased deep tissue infection risk. With our pin-site care protocol and detailed guidance in the discharge instructions, patients with pin-track infections received timely and successful treatment with oral antibiotics. Thus, we believe that this is a ‘local problem’ rather than an obstacle or a true complication. Moreover, the cumbersome nature of the external fixator is another drawback, leading to inconvenience in daily activities, and an additional procedure to change the fixation has a tendency to occur in some non-compliant patients. Therefore, we believe a mature discussion of the risks and benefits with patients is necessary; this may decrease the incidence of secondary operations to change the fixation system.

It should be noted that a novel Ni-Ti alloy (the ASC) was used for limited internal fixation in our study. In the past decade, ASCs have been increasingly used in the clinical treatment of various musculoskeletal diseases due to their excellent wear and corrosion resistance, good biocompatibility, and shape memory effect [19–22]. According to our study, we believe that this connector is also a practical internal apparatus for treating open tibial shaft fractures. It can be used in conditions that are not suitable to be enhanced with cortical screws, including transverse fractures, fragments without the enough opposite cortex, and friable fragments under twisting force. Furthermore, this connector is much more flexible, which helps find the proper placement position for sufficient soft tissue coverage, as well as for procedures in narrow spaces, without additional damage to surrounding tissues. In addition, due to the inherent property of the shape memory alloy, the compression arms in the device provide sustained axial compression forces that can be transmitted across the fracture site to accelerate fracture healing [19–22, 28].

There are several limitations to this study. The study was retrospective and non-randomized. Moreover, the sample size, while not small, was not large enough for more complex methods to control for confounding (subgroup analysis, multivariate analysis, etc.). Additionally, we did not routinely perform a functional score or life-quality score during follow-up, losing the relevant information in the treatment by the two therapies. To that end, the definition of union was not well-defined. In addition, it should be noted that the cost data were simply the direct costs of hospitalization. Finally, although this preliminary study revealed the feasibility of using ASCs in open tibial diaphyseal fractures, further biomechanical studies and analyses are needed.

Despite these limitations, our conclusions were strengthened by several study design characteristics. This was a retrospective cohort study comparing definitive external fixation using a conventional frame to external fixation plus limited internal fixation for open tibial shaft fractures. To minimize selection bias, as well as results bias, we excluded a portion of the patients (please refer to the exclusion criteria in the Patients and Methods section). Additionally, this study contained a reasonable sample size (*n* = 152) and provided a somewhat novel and effective approach in treating such fractures. Moreover, the study also achieved a minimum follow-up of more than 1 year.

## Conclusions

In the current study, limited internal fixation combined with external fixation served as a reliable means compared with simple external fixation for managing open tibial shaft fractures. The additional internal implants did not significantly increase the risk of infection. In contrast, the combined fixation technique was beneficial for initial reduction and subsequent fixation stability, thereby permitting early rehabilitation and rapid healing with a low incidence of postoperative complications. However, further prospective comparative studies with larger samples and longer follow-up periods are required to accurately evaluate the clinical outcomes of this combined technique.

## Data Availability

The datasets analysed during the current study are available from the corresponding author upon reasonable request.

## Abbreviations

ASC: the Ni-Ti arched shape-memory connector
EF: external fixation
EF-LIF: external fixation combined with limited internal fixation
LIF: limited internal fixation
